# Fixed Point Theorems for Generalized **α**-**β**-Weakly Contraction Mappings in Metric Spaces and Applications

**DOI:** 10.1155/2014/784207

**Published:** 2014-05-07

**Authors:** Abdul Latif, Chirasak Mongkolkeha, Wutiphol Sintunavarat

**Affiliations:** ^1^Department of Mathematics, King Abdulaziz University, P.O. Box 80203, Jeddah 21589, Saudi Arabia; ^2^Department of Mathematics, Faculty of Liberal Arts and Science, Kasetsart University, Kamphaeng-Saen Campus, Nakhon Pathom 73140, Thailand; ^3^Department of Mathematics and Statistics, Faculty of Science and Technology, Thammasat University Rangsit Center, Pathum Thani 12121, Thailand

## Abstract

We extend the notion of generalized weakly contraction mappings due to Choudhury et al. (2011) to generalized *α*-*β*-weakly contraction mappings. We show with examples that our new class of mappings is a real generalization of several known classes of mappings. We also establish fixed point results for such mappings in metric spaces. Applying our new results, we obtain fixed point results on ordinary metric spaces, metric spaces endowed with an arbitrary binary relation, and metric spaces endowed with graph.

## 1. Introduction

The well-known Banach's contraction principle has been generalized in many ways over the years [1–6]. One of the most interesting studies is the extension of Banach's contraction principle to a case of weakly contraction mappings which was first given by Alber and Guerre-Delabriere [7] in Hilbert spaces. In 2001, Rhoades [8] has shown that the result of Alber and Guerre-Delabriere [7] is also valid in complete metric spaces. Fixed point problems involving weak contractions and mappings satisfying weak contractive type inequalities have been considered in [9–13] and references therein.

On the other hand, the concept of the altering distance function was introduced by Khan et al. [14]. In 2011, Choudhury et al. [15] generalized weakly contraction mappings by using an altering distance control function and proved fixed point theorem for a pair of these mappings. Some generalizations of this function of fixed point problems in metric and probabilistic metric spaces have been studied [16–18].

Recently, Samet et al. [19] introduced the concepts of *α*-*ψ*-contraction mappings and *α*-admissible mappings and established various fixed point theorems for such mappings in complete metric spaces. Afterwards, many fixed point results via the concepts of *α*-admissible mappings occupied a prominent place in many aspects (see [20–25] and references therein).

From the mentioned above, we introduce the concept of generalized *α*-*β*-weakly contraction mappings and give some examples to show the real generality of these mappings. We also obtain fixed point results for such mappings. Our result improves and complements several results in the literatures. As an application of our results, fixed point results on ordinary metric spaces, metric spaces endowed with an arbitrary binary relation, and metric spaces endowed with graph are also derived from our results.

## 2. Preliminaries

In this section, we give some notations and basic knowledge. Throughout this paper, *N* denotes the set of positive integers.

**Definition 1 (see [14])**

A function *ψ* : [0, *∞*)→[0, *∞*) is called an* altering distance function* if the following properties are satisfied:

- *ψ* is monotone increasing and continuous;
- *ψ*(*t*) = 0 if and only if *t* = 0.

**Definition 2 (see [26])**

Let (*X*, *d*) be a metric space and let *T* be a self-mapping on *X*. A mapping *T* is said to be* contraction* if, for each *x*, *y* ∈ *X*, one has

(1) $$\begin{array}{c} d\left(Tx,Ty\right)\leq kd\left(x,y\right), \end{array}$$

where *k* ∈ [0,1).

**Definition 3 (see [8])**

Let (*X*, *d*) be a metric space and let *T* be a self-mapping on *X*. A mapping *T* is said to be* weak contraction* if, for each *x*, *y* ∈ *X*, one has

(2) $$\begin{array}{c} d\left(Tx,Ty\right)\leq d\left(x,y\right)-\phi \left(d\left(x,y\right)\right), \end{array}$$

where *ϕ* : [0, *∞*)→[0, *∞*) is a continuous and nondecreasing function such that *ϕ*(*t*) = 0 if and only if *t* = 0.

In fact, if we take *ϕ*(*t*) = (1 − *k*)*t* for all *t* ≥ 0, where 0 ≤ *k* < 1, then the condition (2) becomes (1).

In 2011, Choudhury et al. [15] introduced the concept of a generalized weakly contractive condition as follows.

**Definition 4 (see [15])**

Let (*X*, *d*) be a metric space and let *T* be a self-mapping on *X*. A mapping *T* is said to be a* generalized weakly contraction*, if, for each *x*, *y* ∈ *X*, one has

(3) ψ(d(Tx,Ty))≤ψ(m(x,y))− ϕ(max⁡⁡{d(x,y),d(y,Ty)}),

where

(4) m(x,y)=max⁡⁡{d(x,y),d(x,Tx),d(y,Ty),     12(d(x,Ty)+d(y,Tx))},

*ψ* : [0, *∞*)→[0, *∞*) is altering distance function, and *ϕ* : [0, *∞*)→[0, *∞*) is a continuous function with *ϕ*(*t*) = 0 if and only if *t* = 0.

**Remark 5**

It is easy to see that a generalized weakly contractive condition (3) is more general than several generalized contractive conditions. The following conditions are an example of a special case of a generalized weakly contractive condition (3):

- *d*(*Tx*, *Ty*) ≤ *km*(*x*, *y*) for all *x*, *y* ∈ *X*, where *k* ∈ [0,1);
- *d*(*Tx*, *Ty*) < *m*(*x*, *y*) for all *x*, *y* ∈ *X*;
- *d*(*Tx*, *Ty*) ≤ *m*(*x*, *y*) − (1 − *k*)max⁡⁡{*d*(*x*, *y*), *d*(*y*, *Ty*)} for all *x*, *y* ∈ *X*, where *k* ∈ [0,1).

Moreover, the contractive condition (1) is also a special case of condition (3).

**Definition 6 (see [19])**

Let *X* be a nonempty set and let *α* : *X* × *X* → [0, *∞*) be a mapping. A self-mapping *T* : *X* → *X* is said to be *α-admissible* if the following condition holds:

(5) $$\begin{array}{c} x,y\in X,\;\alpha \left(x,y\right)\geq 1⟹\alpha \left(Tx,Ty\right)\geq 1. \end{array}$$

**Example 7 (see [19])**

Let *X* = [0, *∞*) and define *T* : *X* → *X* and *α* : *X* × *X* → [0, *∞*) by

(6) $$\begin{array}{c} Tx=\sqrt{x},\;\forall x\in X,\\ \alpha \left(x,y\right)=\{\begin{array}{cc} e^{x-y}; & x\geq y,\\ 0; & x<y. \end{array} \end{array}$$

Then *T* is *α*-admissible.

## 3. Main Results

In this section, we introduce the concept of generalized *α*-*β*-weakly contraction mappings and prove the fixed point theorems for such mappings.

**Definition 8**

Let (*X*, *d*) be a metric space, *α*, *β* : *X* × *X* → [0, *∞*) two given mappings, and *T* a self-mapping on *X*. A mapping *T* is said to be a* generalized α*-*β*-*weakly contraction type A* if, for each *x*, *y* ∈ *X*, one has

(7) ψ(d(Tx,Ty))≤β(x,y)ψ(m(x,y)) −α(x,y)ϕ(max⁡⁡{d(x,y),d(y,Ty)}),

where

(8) m(x,y)=max⁡⁡{d(x,y),d(x,Tx),d(y,Ty),     12(d(x,Ty)+d(y,Tx))},

*ψ* : [0, *∞*)→[0, *∞*) is altering distance function, and *ϕ* : [0, *∞*)→[0, *∞*) is a continuous function with *ϕ*(*t*) = 0 if and only if *t* = 0.

**Definition 9**

Let (*X*, *d*) be a metric space, *α*, *β* : *X* × *X* → [0, *∞*) two given mappings, and *T* a self-mapping on *X*. A mapping *T* is said to be a* generalized α*-*β*-*weakly contraction type B* if, for each *x*, *y* ∈ *X*, one has

(9) α(x,y)ψ(d(Tx,Ty))≤β(x,y)ψ(m(x,y)) −ϕ(max⁡⁡{d(x,y),d(y,Ty)}),

where

(10) m(x,y)=max⁡{d(x,y),d(x,Tx),d(y,Ty),     12(d(x,Ty)+d(y,Tx))},

*ψ* is altering distance function, and *ϕ* : [0, *∞*)→[0, *∞*) is a continuous function with *ϕ*(*t*) = 0 if and only if *t* = 0.

If we take *α*(*x*, *y*) = *β*(*x*, *y*) = 1 for all *x*, *y* ∈ *X*, then generalized *α*-*β*-weakly contraction mappings type *A* and type *B* become generalized weakly contraction mappings due to Choudhury et al. [15]. Therefore, classes of generalized *α*-*β*-weakly contraction mappings type *A* and type *B* are larger than the class of generalized weakly contraction mappings. Next, we give some examples to show the real generality of classes of generalized *α*-*β*-weakly contraction mappings.

**Example 10**

Let *X* = [0,1]∪{2,3, 4,…}. From [27], *X* is a complete metric space with metric defined by

(11) $$\begin{array}{c} d\left(x,y\right)=\{\begin{array}{cc} \left|x-y\right|; & x,y\in \left[0,1\right],\\ x+y; & \text{one}\;\text{of}\;\;x,y\notin \left[0,1\right],\;\;x\neq y,\\ 0; & x=y. \end{array} \end{array}$$

Let a mapping *T* : *X* → *X* be defined by

(12) $$\begin{array}{c} Tx=\{\begin{array}{cc} \frac{x}{2}; & x\in \left[0,1\right],\\ x-1; & x\in \left\{2,3,4,\ldots ,10\right\},\\ x^{2}; & x\in \left\{11,12,13,\ldots \right\}. \end{array} \end{array}$$

First, we show that *T* is generalized *α*-*β*-weakly contractive type *A* with the functions *ψ*, *ϕ* : [0, *∞*)→[0, *∞*) and *α*, *β* : *X* × *X* → [0, *∞*) defined by

(13)   ψ(t)={t;0≤t≤1,t2;t>1,  ϕ(t)={t2;0≤t≤1,12;t>1,α(x,y)={1;x,y∈[0,1],2;one  of  x,y∉[0,1],  x,y≤10,0;otherwise,β(x,y) ={1;x,y∈[0,1]∪{2,3,4,…,10},ψ(d(Tx,Ty));otherwise.

Next we show that *T* is a generalized *α*-*β*-weakly contraction mapping type *A*. For *x*, *y* ∈ *X*, we distinguish the following cases.

**Case 1 (*x*, *y* ∈ [0,1])**

Without loss of generality, we may assume that *x* ≥ *y*. Now we obtain that

(14) m(x,y)=max⁡⁡{d(x,y),d(x,Tx),d(y,Ty),     12(d(x,Ty)+d(y,Tx))}=max⁡{x−y,x2,y2,12(x−y2+|y−x2|)}={x−y;0≤y≤x2,x2;x2<y≤x.

In case of 0 ≤ *y* ≤ *x*/2, we have *ϕ*(max⁡⁡{*d*(*x*, *y*), *d*(*y*, *Ty*)}) = (1/2)(*x* − *y*) and then

(15) ψ(d(Tx,Ty))=12(x−y)=(x−y)−12(x−y)=(x−y)−ϕ(max⁡{d(x,y),d(y,Ty)})≤β(x,y)ψ(m(x,y)) −α(x,y)ϕ(max⁡⁡{d(x,y),d(y,Ty)}).

In case of *x*/2 < *y* ≤ *x*, we have max⁡⁡{*d*(*x*, *y*), *d*(*y*, *Ty*)} = max⁡⁡{*x* − *y*, *y*/2}.

If max⁡⁡{*d*(*x*, *y*), *d*(*y*, *Ty*)} = *y*/2, then we have

(16) ψ(d(Tx,Ty))=12(x−y)≤x2−y4=β(x,y)ψ(m(x,y)) −α(x,y)ϕ(max⁡⁡{d(x,y),d(y,Ty)}).

If max⁡⁡{*d*(*x*, *y*), *d*(*y*, *Ty*)} = *x* − *y*, then we have

(17) ψ(d(Tx,Ty))=12(x−y)<x2−x4≤x4<y2=x2−12(x−y)=β(x,y)ψ(m(x,y)) −α(x,y)ϕ(max⁡⁡{d(x,y),d(y,Ty)}).

Therefore, for *x*, *y* ∈ [0,1], we get that *T* satisfies condition (7).

**Case 2 (*x* ∈ {2,3, 4,…, 10} and *y* ∈ [0,1])**

In this case, we obtain that

(18) $$\begin{array}{c} -x^{2}+xy<0,\;-x^{2}+\left(\frac{y^{2}}{4}-y\right)<0,\;-x^{2}+1<0, \end{array}$$

(19) m(x,y)=max⁡⁡{d(x,y),d(x,Tx),d(y,Ty),     12(d(x,Ty)+d(y,Tx))}=max⁡⁡{x+y,2x−1,3y2,12(x+y2+y+x−1)}=max⁡⁡{x+y,2x−1,3y2,12(2x+3y2−1)}=2x−1.

From (18), we obtain that

(20) ψ(d(Tx,Ty))=ψ(x−1+y2)=(x+y2−1)2=x2+xy+y24−2x−y+1=x2+xy+y24−2x−y+1+3x2−3x2=4x2+(−x2+xy)+(−x2+y24−y) +(−x2+1)−2x≤4x2−2x=4x2−2x+1−1=(2x−1)2−(2)(12)=β(x,y)ψ(m(x,y)) −α(x,y)ϕ(max⁡⁡{d(x,y),d(y,Ty)}).

Therefore, we conclude that *T* satisfies condition (7) in this case.

**Case 3 (*x* ∈ [0,1] and *y* ∈ {2,3, 4,…, 10})**

This case is similar to Case 2.

**Case 4 (*x* ∈ {2,3, 4,…, 10} and *y* ∈ {2,3, 4,…, 10})**

Now we obtain that

(21) m(x,y)=max⁡⁡{d(x,y),d(x,Tx),d(y,Ty),       12(d(x,Ty)+d(y,Tx))}={max⁡{0,2x−1,2y−1,    12(x+y−1+y+x−1)};x=y,max⁡{x+y,2x−1,2y−1,    12(x+y−1+y+x−1)};x≠y.={2x−1;x=y,max⁡⁡{x+y,2x−1,2y−1,     x+y−1};x≠y.={2x−1;y≤x,2y−1;y>x.

If *x* = *y*, we have *ϕ*(max⁡⁡{*d*(*x*, *y*), *d*(*y*, *Ty*)}) = 2*x* − 1 and so

(22) ψ(d(Tx,Ty))=ψ(0)=0≤(2x−1)2−2(12)=ψ(2x−1)−α(x,y)ϕ(2x−1)=β(x,y)ψ(m(x,y)) −α(x,y)ϕ(max⁡⁡{d(x,y),d(y,Ty)}).

If *y* < *x*, we have *ϕ*(max⁡⁡{*d*(*x*, *y*), *d*(*y*, *Ty*)}) = 2*x* − 1, and hence

(23) ψ(d(Tx,Ty))=ψ(x+y−2)=(x+y−2)2<(2x−2)2=(2x−1)2+(3−4x)<(2x−1)2−1=(2x−1)2−2(12)=ψ(2x−1)−α(x,y)ϕ(2x−1)=β(x,y)ψ(m(x,y)) −α(x,y)ϕ(max⁡⁡{d(x,y),d(y,Ty)}).

If *y* > *x*, we have *ϕ*(max⁡⁡{*d*(*x*, *y*), *d*(*y*, *Ty*)}) = 2*y* − 1, and hence

(24) ψ(d(Tx,Ty))=ψ(x+y−2)=(x+y−2)2<(2y−2)2=(2y−1)2+(3−4y)<(2y−1)2−1=(2y−1)2−2(12)=ψ(2y−1)−α(x,y)ϕ(2y−1)=β(x,y)ψ(m(x,y)) −α(x,y)ϕ(max⁡⁡{d(x,y),d(y,Ty)}).

Now we conclude that *T* satisfies condition (7) in this case.

**Case 5 (one of *x*, *y* ∉ [0,1]∪{2,3, 4,…, 10})**

If *x* = *y*, we obtain that

(25) ψ(d(Tx,Ty))=ψ(0)=0=ψ(d(Tx,Ty))ψ(m(x,y)) −α(x,y)ϕ(max⁡⁡{d(x,y),d(y,Ty)})=β(x,y)ψ(m(x,y)) −α(x,y)ϕ(max⁡⁡{d(x,y),d(y,Ty)}).

If *x* ≠ *y*, we get *ψ*(*m*(*x*, *y*)) ≥ 1 and so

(26) ψ(d(Tx,Ty))≤ψ(d(Tx,Ty))ψ(m(x,y))=β(x,y)ψ(m(x,y)) −α(x,y)ϕ(max⁡⁡{d(x,y),d(y,Ty)}).

Now we conclude that *T* satisfies condition (7) in this case.

From all cases, we get that *T* is generalized *α*-*β*-weakly contraction mapping type *A*.

**Remark 11**

From Example 10, we can see that *T* is not a generalized weakly contraction mapping. Indeed, putting *x* = 11 and *y* = 12, we get

(27) m(11,12)=max⁡{d(11,12),d(11,T(11)),d(12,T(12)),12(d(11,T(12))+d(12,T(11)))}=156,

(28) ψ(d(T(11),T(12)))=ψ(121+144)=1652>1562>1562−12=ψ(m(11,12))−ϕ(max⁡⁡{d(11,12),d(11,T(12))}).

Before presenting the main results in this paper, we introduce the following concept, which will be used in our results.

**Definition 12**

Let *X* be a nonempty set and *β* : *X* × *X* → [0, *∞*). A self-mapping *T* : *X* → *X* is said to be *β*_0_ *-subadmissible* if the following condition holds:

(29) $$\begin{array}{c} x,y\in X,\;0<\beta \left(x,y\right)\leq 1⟹0<\beta \left(Tx,Ty\right)\leq 1. \end{array}$$

**Definition 13**

Let *X* be a nonempty set and *α* : *X* × *X* → [0, *∞*) a mapping.

- *α* is said to be* forward transitive * if for each *x*, *y*, *z* ∈ *X* for which *α*(*x*, *y*) ≥ 1 and *α*(*y*, *z*) ≥ 1 one has *α*(*x*, *z*) ≥ 1;
- *α* is said to be 0*-backward transitive * if for each *x*, *y*, *z* ∈ *X* for which 0 < *α*(*x*, *y*) ≤ 1 and 0 < *α*(*y*, *z*) ≤ 1 one has 0 < *α*(*x*, *z*) ≤ 1.

### 3.1. Generalized *α*-*β*-Weakly Contraction Mappings Type*A*

In this subsection, we give the fixed point results for generalized *α*-*β*-weakly contraction mappings type *A*.

**Theorem 14**

Let (*X*, *d*) be a complete metric space, *α*, *β* : *X* × *X* → [0, *∞*) two given mappings, and *T* : *X* → *X* a generalized *α*-*β*-weakly contraction type *A*; then the following conditions hold:

(a) *T* is continuous;
(b) *T* is *α*-admissible and *β*_0_-subadmissible;
(c) *α* is forward transitive and *β* is 0-backward transitive;
(d) there exists *x*_0_ ∈ *X* such that 0 < *β*(*x*_0_, *Tx*_0_) ≤ 1 ≤ *α*(*x*_0_, *Tx*_0_).

Then *T* has a fixed point in *X*.

**Proof**

Starting from *x*_0_ ∈ *X* in assumption (d) and letting *x*_*n*+1_ = *Tx*_*n*_ for all *n* ∈ *N* ∪ {0}, if there exists *n*_0_ ∈ *N* ∪ {0} such that *x*_*n*_0__ = *x*_*n*_0_+1_, then *x*_*n*_0__ is a fixed point of *T*. This finishes the proof. Therefore, we may assume that *x*_*n*_ ≠ *x*_*n*+1_ for all *n* ∈ *N* ∪ {0}. Since 0 < *β*(*x*_0_, *Tx*_0_) ≤ 1 ≤ *α*(*x*_0_, *Tx*_0_), we get

(30) $$\begin{array}{c} 0<\beta \left(x_{0},x_{1}\right)\leq 1\leq \alpha \left(x_{0},x_{1}\right). \end{array}$$

It follows from *T* is *α*-admissible and *β*_0_-subadmissible that

(31) $$\begin{array}{c} 0<\beta \left(Tx_{0},Tx_{1}\right)\leq 1\leq \alpha \left(Tx_{0},Tx_{1}\right), \end{array}$$

and then

(32) $$\begin{array}{c} 0<\beta \left(x_{1},x_{2}\right)\leq 1\leq \alpha \left(x_{1},x_{2}\right). \end{array}$$

By repeating this process, we get that {*x*_*n*_} is a sequence in *X* such that *x*_*n*+1_ = *Tx*_*n*_ and

(33) $$\begin{array}{c} 0<\beta \left(x_{n},x_{n+1}\right)\leq 1\leq \alpha \left(x_{n},x_{n+1}\right), \end{array}$$

for all *n* ∈ *N* ∪ {0}. By using the generalized *α*-*β*-weakly contractive condition type *A* of *T*, we have

(34) ψ(d(xn+1,xn+2))  =ψ(d(Txn,Txn+1))  ≤β(xn,xn+1)ψ(m(xn,xn+1))−α(xn,xn+1)   ×ϕ(max⁡⁡{d(xn,xn),d(xn+1,Txn+1)})  ≤ψ(m(xn,xn+1))   −ϕ(max⁡⁡{d(xn,xn+1),d(xn+1,xn+2)}),

for all *n* ∈ *N* ∪ {0}. Now we obtain that

(35) m(xn,xn+1) =max⁡{d(xn,xn+1),d(xn,Txn),d(xn+1,Txn+1),       12(d(xn,Txn+1)+d(xn+1,Txn))} =max⁡{d(xn,xn+1),d(xn,xn+1),d(xn+1,xn+2),       12(d(xn,xn+2)+d(xn+1,xn+1))} =max⁡{d(xn,xn+1),d(xn,xn+1),d(xn+1,xn+2),       12(d(xn,xn+2))} ≤max⁡{d(xn,xn+1),d(xn+1,xn+2)},

for all *n* ∈ *N* ∪ {0}. From (34) and (35), we get

(36) ψ(d(xn+1,xn+2)) ≤ψ(max⁡⁡{d(xn,xn+1),d(xn+1,xn+2)})  −ϕ(max⁡⁡{d(xn,xn+1),d(xn+1,xn+2)}),

for all *n* ∈ *N* ∪ {0}.

Suppose that *d*(*x*_*n*_, *x*_*n*+1_) ≤ *d*(*x*_*n*+1_, *x*_*n*+2_) for some *n* ∈ *N* ∪ {0}. Then we have

(37) ψ(d(xn+1,xn+2))≤ψ(d(xn+1,xn+2)) −ϕ(d(xn+1,xn+2))<ψ(d(xn+1,xn+2))

which is a contradiction. Hence *d*(*x*_*n*+1_, *x*_*n*+2_) < *d*(*x*_*n*_, *x*_*n*+1_) for all *n* ∈ *N* ∪ {0}. This means that {*d*(*x*_*n*_, *x*_*n*+1_)} is a monotone decreasing sequence. Since {*d*(*x*_*n*_, *x*_*n*+1_)} is bounded below, there exists *r* ≥ 0 such that

(38) $$\begin{array}{c} \underset{n\to \infty }{\lim}d\left(x_{n},x_{n+1}\right)=r. \end{array}$$

Using (36), we get

(39) ψ(d(xn+1,xn+2))≤ψ(d(xn,xn+1)) −ϕ(d(xn,xn+1)),

for all *n* ∈ *N* ∪ {0}. Taking *n* → *∞* in the above inequality, we have

(40) $$\begin{array}{c} \psi \left(r\right)\leq \psi \left(r\right)-\phi \left(r\right). \end{array}$$

This implies that *r* = 0; that is,

(41) $$\begin{array}{c} \underset{n\to \infty }{\lim}d\left(x_{n},x_{n+1}\right)=0. \end{array}$$

Next, we will prove that {*x*_*n*_} is a Cauchy sequence. Suppose that {*x*_*n*_} is not a Cauchy sequence. Then there exists *ε* > 0 such that

(42) $$\begin{array}{c} d\left(x_{m_{k}},x_{n_{k}}\right)\geq \varepsilon \end{array}$$

for all *n*_*k*_ > *m*_*k*_ ≥ *k*, where *k* ∈ *N*. Further, corresponding to *m*_*k*_, we can choose *n*_*k*_ in such a way that it is the smallest integer with *n*_*k*_ > *m*_*k*_ ≥ *k* satisfying (42). Then we have

(43) $$\begin{array}{c} d\left(x_{m_{k}},x_{n_{k}}\right)\geq \varepsilon ,\;\;d\left(x_{m_{k}},x_{n_{k}-1}\right)<\varepsilon . \end{array}$$

By using (43) and triangular inequality, we get

(44) ε≤d(xmk,xnk)≤d(xmk,xnk−1)+d(xnk−1,xnk)≤ε+d(xnk−1,xnk).

From (41) and (44), we have

(45) $$\begin{array}{c} \underset{k\to \infty }{\lim}d\left(x_{m_{k}},x_{n_{k}}\right)=\varepsilon . \end{array}$$

From the triangular inequality, we get

(46) d(xmk,xnk)≤d(xmk,xmk+1)+d(xmk+1,xnk+1) +d(xnk+1,xnk)=d(xmk+1,xnk+1)+d(xmk,xmk+1) +d(xnk+1,xnk)≤d(xmk+1,xmk)+d(xmk,xnk) +d(xnk,xnk+1) +d(xmk,xmk+1)+d(xnk+1,xnk)≤2d(xmk+1,xmk)+d(xmk,xnk) +2d(xnk,xnk+1).

Using (45) and (46), we get

(47) $$\begin{array}{c} \underset{k\to \infty }{\lim}d\left(x_{m_{k}+1},x_{n_{k}+1}\right)=\varepsilon . \end{array}$$

Again, by the triangular inequality, we get

(48) $$\begin{array}{c} d\left(x_{m_{k}},x_{n_{k}}\right)\leq d\left(x_{m_{k}},x_{n_{k}+1}\right)+d\left(x_{n_{k}+1},x_{n_{k}}\right),\\ d\left(x_{m_{k}},x_{n_{k}+1}\right)\leq d\left(x_{m_{k}},x_{n_{k}}\right)+d\left(x_{n_{k}},x_{n_{k}+1}\right). \end{array}$$

Using (48), we obtain that

(49) $$\begin{array}{c} \underset{k\to \infty }{\lim}d\left(x_{m_{k}},x_{n_{k}+1}\right)=\varepsilon . \end{array}$$

Similarly, we can prove

(50) $$\begin{array}{c} \underset{k\to \infty }{\lim}d\left(x_{n_{k}},x_{m_{k}+1}\right)=\varepsilon . \end{array}$$

Since *α* is forward transitive, *β* is 0-backward transitive, and *n*_*k*_ > *m*_*k*_, we can conclude that

(51) $$\begin{array}{c} 0<\beta \left(x_{m_{k}},x_{n_{k}}\right)\leq 1\leq \alpha \left(x_{m_{k}},x_{n_{k}}\right). \end{array}$$

In view of the fact that *T* is generalized *α*-*β*-weakly contractive type *A* mapping and (51), we have

(52) ψ(d(xmk+1,xnk+1)) =ψ(d(Txmk,Txnk)) ≤β(xmk,xnk)ψ(m(xmk,xnk))  −α(xmk,xnk)ϕ(max⁡⁡{d(xmk,xnk),d(xnk,Txnk)}) ≤ψ(m(xmk,xnk))  −ϕ(max⁡⁡{d(xmk,xnk),d(xnk,Txnk)}) ≤ψ(max⁡{d(xmk,xnk),d(xmk,Txmk),d(xnk,Txnk),         12(d(xmk,Txnk)+d(xnk,Txmk))})  −ϕ(max⁡⁡{d(xmk,xnk),d(xnk,Txnk)}) =ψ(max⁡⁡{d(xmk,xnk),d(xmk,xmk+1),d(xnk,xnk+1),⁡         12(d(xmk,xnk+1)+d(xnk,xmk+1))})  −ϕ(max⁡⁡{d(xmk,xnk),d(xnk,xnk+1)}).

Letting *k* → *∞*, by using (45), (47), (49), and (50), we obtain that

(53) $$\begin{array}{c} \psi \left(\varepsilon \right)\leq \psi \left(\varepsilon \right)-\phi \left(\varepsilon \right)<\psi \left(\varepsilon \right) \end{array}$$

which is a contradiction. Then, we deduce that {*x*_*n*_} is a Cauchy sequence. Since *X* is a complete metric space, then there exists x* ∈ *X* such that *x*_*n*_ → x* as *n* → *∞*. From the continuity of *T*, it follows that

(54) $$\begin{array}{c} \underset{n\to \infty }{\lim}x_{n+1}=\underset{n\to \infty }{\lim}Tx_{n}=Tx^{*}. \end{array}$$

Using the uniqueness of limit of the sequence, we conclude that Tx* = x* and the proof is complete.

In the next theorem, the continuity of a generalized *α*-*β*-weakly contraction mapping type *A* in Theorem 14 is replaced by the following condition:

(*C*) if {*x*_*n*_} is a sequence in *X* such that 0 < *β*(*x*_*n*_, *x*_*n*+1_) ≤ 1 ≤ *α*(*x*_*n*_, *x*_*n*+1_) for all *n* ∈ *N* and *x*_*n*_ → *x* as *n* → *∞*, then 0 < *β*(*x*_*n*_, *x*) ≤ 1 ≤ *α*(*x*_*n*_, *x*) for all *n* ∈ *N*.

**Theorem 15**

Let (*X*, *d*) be a complete metric space, *α*, *β* : *X* × *X* → [0, *∞*) two given mappings, and *T* : *X* → *X* a generalized *α*-*β*-weakly contraction type *A*; then the following conditions hold:

(a) condition (*C*) holds;
(b) *T* is *α*-admissible and *β*_0_-subadmissible;
(c) *α* is forward transitive and *β* is 0-backward transitive;
(d) there exists *x*_0_ ∈ *X* such that 0 < *β*(*x*_0_, *Tx*_0_) ≤ 1 ≤ *α*(*x*_0_, *Tx*_0_).

Then *T* has a fixed point in *X*.

**Proof**

As in the proof of Theorem 14, we can find a sequence {*x*_*n*_} in *X* such that *x*_*n*+1_ = *Tx*_*n*_ for all *n* ∈ *N* ∪ {0} and {*x*_*n*_} is Cauchy sequence which converges to some point x* in *X*. Moreover, we have

(55) $$\begin{array}{c} 0<\beta \left(x_{n},x_{n+1}\right)\leq 1\leq \alpha \left(x_{n},x_{n+1}\right), \end{array}$$

for all *n* ∈ *N*. By using condition (*C*), we obtain that

(56) $$\begin{array}{c} 0<\beta \left(x_{n},x\right)\leq 1\leq \alpha \left(x_{n},x\right), \end{array}$$

for all *n* ∈ *N*. Now, let us claim that Tx* = x*. Supposing the contrary, from the fact that *T* is a generalized *α*-*β*-weakly contractive type *A* and (55), we get

(57) ψ(d(xn+1,Tx∗))  =ψ(d(Txn,Tx∗))  ≤β(xn,x∗)ψ(m(xn,x∗))   −α(xn,x∗)ϕ(max⁡⁡{d(xn,x∗),d(x∗,Tx∗)})  ≤β(xn,x∗)ψ(m(xn,x∗))   −ϕ(max⁡⁡{d(xn,x∗),d(x∗,Tx∗)})  ≤ψ(m(xn,x∗))   −ϕ(max⁡⁡{d(xn,x∗),d(x∗,Tx∗)}).

On the other hand, we obtain that

(58) m(xn,x∗)=max⁡{d(xn,x∗),d(xn,Txn),d(x∗,Tx∗),     12(d(xn,Tx∗)+d(x∗,Txn))}=max⁡{d(xn,x∗),d(xn,xn+1),d(x∗,Tx∗),     12(d(xn,Tx∗)+d(x∗,xn+1))},

for all *n* ∈ *N*. Letting *n* → *∞* in (57), by using (58) and the continuity of *ψ*, we get

(59) ψ(d(x∗,Tx∗))≤ψ(d(x∗,Tx∗)) −ϕ(d(x∗,Tx∗))<ψ(d(x∗,Tx∗))

which is a contradiction. Therefore Tx* = x* and the proof is complete.

We obtain that Theorems 14 and 15 cannot claim the uniqueness of fixed point. To assure the uniqueness of the fixed point, we will add the following condition:

(*C*′) for all *x*, *y* ∈ *X* there exists *z* ∈ *X* such that
  (60) $$\begin{array}{c} 0<\beta \left(x,z\right)\leq 1\leq \alpha \left(x,z\right),\\ 0<\beta \left(y,z\right)\leq 1\leq \alpha \left(y,z\right). \end{array}$$

**Definition 16**

Let *X* be a nonempty set, *T* : *X* → *X* a mapping, and *x*_0_ ∈ *X*. The orbit of *T* at *x*_0_ is denoted by *O*(*T*, *x*_0_) and defined by

(61) $$\begin{array}{c} O\left(T,x_{0}\right):=\left\{x_{0},T\left(x_{0}\right),T^{2}\left(x_{0}\right),T^{3}\left(x_{0}\right),\ldots ,T^{n}\left(x_{0}\right),\ldots \right\}, \end{array}$$

where *T* ^*n*+1^ *x*_0_ = *T*(*T* ^*n*^ *x*_0_) and *T* ^0^ *x*_0_ = *x*_0_.

**Theorem 17**

By adding condition (*C*′) to the hypotheses of Theorem 14 (or Theorem 15) and the limit of orbit *O*(*T*, *z*) exists, where *z* is an element in *X* satisfying (60). Then *T* has a unique fixed point.

**Proof**

Suppose that *x* and x* are two fixed points of *T*. By condition (*C*′) there exists *z* ∈ *X* such that

(62) $$\begin{array}{c} 0<\beta \left(x,z\right)\leq 1\leq \alpha \left(x,z\right),\\ 0<\beta \left(x^{*},z\right)\leq 1\leq \alpha \left(x^{*},z\right). \end{array}$$

It follows from *T* is *α*-admissible and *β*_0_-subadmissible that

(63) $$\begin{array}{c} 0<\beta \left(x,T^{n}z\right)\leq 1\leq \alpha \left(x,T^{n}z\right),\\ 0<\beta \left(x^{*},T^{n}z\right)\leq 1\leq \alpha \left(x^{*},T^{n}z\right), \end{array}$$

for all *n* ∈ *N*. Since the limit of *O*(*T*, *z*) exists, we get that {*T* ^*n*^ *z*} converges to some element in *X*. Let us claim that *T* ^*n*^ *z* → *x* as *n* → *∞*. Suppose the contrary; that is

(64) $$\begin{array}{c} \underset{n\to \infty }{\lim}d\left(T^{n}z,x\right):=r>0. \end{array}$$

By the generalized *α*-*β*-weakly contractive condition type *A* of *T*, we have

(65) ψ(d(x,Tn+1z))  =ψ(d(Tx,T(Tnz)))  ≤β(x,Tnz)ψ(m(x,Tnz))−α(x,Tnz)   ×ϕ(max⁡⁡{d(x,Tnz),d(Tnz,T(Tnz))})  ≤β(x,Tnz)ψ(m(x,Tnz))   −ϕ(max⁡⁡{d(x,Tnz),d(Tnz,T(Tnz))})  ≤ψ(m(x,Tnz))   −ϕ(max⁡{d(x,Tnz),d(Tnz,Tn+1z)}),

for all *n* ∈ *N*. On the other hand, we have

(66) m(x,Tnz)=max⁡{d(x,Tnz),d(x,Tx),d(Tnz,T(Tnz)),     12(d(x,T(Tnz))+d(Tnz,Tx))}=max⁡⁡{d(x,Tnz),d(Tnz,Tn+1z),     12(d(x,Tn+1z)+d(Tnz,x))},

for all *n* ∈ *N*. From (64), (65), (66), and the property of *ψ* and *ϕ*, we obtain that

(67) $$\begin{array}{c} \psi \left(r\right)\leq \psi \left(r\right)-\phi \left(r\right)<\psi \left(r\right) \end{array}$$

which is a contradiction, and hence *T* ^*n*^ *z* → *x* as *n* → *∞*. Similarly, we can show that *T* ^*n*^ *z* → x* as *n* → *∞*. Using the uniqueness of limit of the sequence, we conclude that *x* = x* and the proof is complete.

### 3.2. Generalized *α*-*β*-Weakly Contraction Mappings Type *B*

In this subsection, we obtain the existence and uniqueness of fixed point theorems for generalized *α*-*β*-weakly contraction mappings type *B*.

**Theorem 18**

Let (*X*, *d*) be a complete metric space, *α*, *β* : *X* × *X* → [0, *∞*) two given mappings, and *T* : *X* → *X* a generalized *α*-*β*-weakly contraction type *B*; then the following conditions hold:

(a) *T* is continuous;
(b) *T* is *α*-admissible and *β*_0_-subadmissible;
(c) *α* is forward transitive and *β* is 0-backward transitive;
(d) there exists *x*_0_ ∈ *X* such that 0 < *β*(*x*_0_, *Tx*_0_) ≤ 1 ≤ *α*(*x*_0_, *Tx*_0_).

Then *T* has a fixed point in *X*.

**Proof**

As in the proof of Theorem 14, we can find a sequence {*x*_*n*_} in *X* such that *x*_*n*+1_ = *Tx*_*n*_, *x*_*n*_ ≠ *x*_*n*+1_, and

(68) $$\begin{array}{c} 0<\beta \left(x_{n},x_{n+1}\right)\leq 1\leq \alpha \left(x_{n},x_{n+1}\right), \end{array}$$

for all *n* ∈ *N* ∪ {0}. Moreover, for each *n* ∈ *N* ∪ {0}, we have

(69) $$\begin{array}{c} m\left(x_{n},x_{n+1}\right)\leq \max\left\{d\left(x_{n},x_{n+1}\right),d\left(x_{n+1},x_{n+2}\right)\right\}. \end{array}$$

Since *T* is a generalized *α*-*β*-weakly contraction mapping type *B*, for each *n* ∈ *N* ∪ {0}, we get

(70) ψ(d(xn+1,xn+2))  =ψ(d(Txn,Txn+1))  ≤α(xn,xn+1)ψ(d(Txn,Txn+1))  ≤β(xn,xn+1)ψ(m(xn,xn+1))   −ϕ(max⁡⁡{d(xn,xn),d(xn+1,xn+2)})  ≤ψ(m(xn,xn+1))   −ϕ(max⁡⁡{d(xn,xn+1),d(xn+1,xn+2)})  ≤ψ(max⁡⁡{d(xn,xn+1),d(xn+1,xn+2)})   −ϕ(max⁡⁡{d(xn,xn+1),d(xn+1,xn+2)}).

Suppose that *d*(*x*_*n*_, *x*_*n*+1_) ≤ *d*(*x*_*n*+1_, *x*_*n*+2_) for some *n* ∈ *N* ∪ {0}. From (70), we get

(71) ψ(d(xn+1,xn+2))≤ψ(d(xn+1,xn+2)) −ϕ(d(xn+1,xn+2))<ψ(d(xn+1,xn+2))

which is a contradiction. Therefore *d*(*x*_*n*+1_, *x*_*n*+2_) < *d*(*x*_*n*_, *x*_*n*+1_) for all *n* ∈ *N* ∪ {0}. This means that {*d*(*x*_*n*_, *x*_*n*+1_)} is a monotone decreasing sequence. It follows from a sequence {*d*(*x*_*n*_, *x*_*n*+1_)} bounded below that there exists *r* ≥ 0 such that

(72) $$\begin{array}{c} \underset{n\to \infty }{\lim}d\left(x_{n},x_{n+1}\right)=r. \end{array}$$

From (70), we get

(73) ψ(d(xn+1,xn+2))≤ψ(d(xn,xn+1)) −ϕ(d(xn,xn+1)),

for all *n* ∈ *N* ∪ {0}. Taking *n* → *∞* in the above inequality, we have

(74) $$\begin{array}{c} \psi \left(r\right)\leq \psi \left(r\right)-\phi \left(r\right). \end{array}$$

This implies that *r* = 0; that is,

(75) $$\begin{array}{c} \underset{n\to \infty }{\lim}d\left(x_{n},x_{n+1}\right)=0. \end{array}$$

Next, we will prove that {*x*_*n*_} is a Cauchy sequence. Suppose that {*x*_*n*_} is not a Cauchy sequence. Then there exists *ε* > 0 such that

(76) $$\begin{array}{c} d\left(x_{m_{k}},x_{n_{k}}\right)\geq \varepsilon , \end{array}$$

for all *n*_*k*_ > *m*_*k*_ ≥ *k*, where *k* ∈ *N*. Further, corresponding to *m*_*k*_, we can choose *n*_*k*_ in such a way that it is the smallest integer with *n*_*k*_ > *m*_*k*_ ≥ *k* satisfying (76). Then we have

(77) $$\begin{array}{c} d\left(x_{m_{k}},x_{n_{k}}\right)\geq \varepsilon ,\;\;d\left(x_{m_{k}},x_{n_{k}-1}\right)<\varepsilon . \end{array}$$

As the same argument in Theorem 14, we have

(78) lim⁡k→∞d(xmk,xnk)=lim⁡n→∞d(xmk,xnk+1)=lim⁡n→∞d(xnk,xmk+1)=ε.

Moreover, we have

(79) $$\begin{array}{c} 0<\beta \left(x_{m_{k}},x_{n_{k}}\right)\leq 1\leq \alpha \left(x_{m_{k}},x_{n_{k}}\right). \end{array}$$

In view of the fact that *T* is generalized *α*-*β*-weakly contraction mapping type *B*, we have

(80) ψ(d(xmk+1,xnk+1)) =ψ(d(Txmk,Txnk)) ≤α(xmk,xnk)ψ(d(xmk+1,xnk+1)) ≤β(xmk,xnk)ψ(m(xmk,xnk))  −ϕ(max⁡⁡{d(xmk,xnk),d(xnk,Txnk)}) ≤ψ(max⁡⁡{d(xmk,xnk),d(xmk,Txmk),d(xnk,Txnk),        12(d(xmk,Txnk)+d(xnk,Txmk))})  −ϕ(max⁡⁡{d(xmk,xnk),d(xnk,Txnk)}) =ψ(max⁡⁡{d(xmk,xnk),d(xmk,xmk+1),d(xnk,xnk+1),        12(d(xmk,xnk+1)+d(xnk,xmk+1))})  −ϕ(max⁡⁡{d(xmk,xnk),d(xnk,xnk+1)}),

for all *k* ∈ *N*. Letting *k* → *∞* in the above relation, we obtain that

(81) $$\begin{array}{c} \psi \left(\varepsilon \right)\leq \psi \left(\varepsilon \right)-\phi \left(\varepsilon \right)<\psi \left(\varepsilon \right) \end{array}$$

which is a contradiction. Therefore, we deduce that {*x*_*n*_} is a Cauchy sequence and so it converges to some element x* ∈ *X*. By the continuity of *T*, we get

(82) $$\begin{array}{c} \underset{n\to \infty }{\lim}x_{n+1}=\underset{n\to \infty }{\lim}Tx_{n}=Tx^{*}, \end{array}$$

and hence Tx* = x*. Therefore the proof is complete.

**Theorem 19**

Let (*X*, *d*) be a complete metric space, *α*, *β* : *X* × *X* → [0, *∞*) two given mappings, and *T* : *X* → *X* a generalized *α*-*β*-weakly contraction type *B*; then the following conditions hold:

(a) condition (*C*) holds;
(b) *T* is *α*-admissible and *β*_0_-subadmissible;
(c) *α* is forward transitive and *β* is 0-backward transitive;
(d) there exists *x*_0_ ∈ *X* such that 0 < *β*(*x*_0_, *Tx*_0_) ≤ 1 ≤ *α*(*x*_0_, *Tx*_0_).

Then *T* has a fixed point in *X*.

**Proof**

As in the proof of Theorem 18, we can find a sequence {*x*_*n*_} in *X* such that *x*_*n*+1_ = *Tx*_*n*_ for all *n* ∈ *N* ∪ {0} and {*x*_*n*_} is a Cauchy sequence converging to some point x* in *X*.

Also, as in the proof of Theorem 15, for each *n* ∈ *N*, we get

(83) $$\begin{array}{c} 0<\beta \left(x_{n},x^{*}\right)\leq 1\leq \alpha \left(x_{n},x^{*}\right), \end{array}$$

(84) $$\begin{array}{c} \underset{n\to \infty }{\lim}m\left(x_{n},x^{*}\right)=d\left(x^{*},Tx^{*}\right). \end{array}$$

Now, let us claim that Tx* = x*. On contrary, assume that x* ≠ Tx* that is *d*(x*, Tx*) ≠ 0. By using (83) and a generalized *α*-*β*-weakly contractive condition type *B*, we get

(85) ψ(d(xn+1,Tx∗))=ψ(d(Txn,Tx∗))≤α(xn,x∗)ψ(d(Txn,Tx∗))≤β(xn,x∗)ψ(m(xn,x∗)) −ϕ(max⁡⁡{d(xn,x∗),d(x∗,Tx∗)})≤ψ(m(xn,x∗)) −ϕ(max⁡⁡{d(xn,x∗),d(x∗,Tx∗)}).

Letting *n* → *∞* in (85), we get

(86) ψ(d(x∗,Tx∗))≤ψ(d(x∗,Tx∗)) −ϕ(d(x∗,Tx∗))<ψ(d(x∗,Tx∗))

which is a contradiction. Therefore Tx* = x* and the proof is complete.

**Theorem 20**

By adding condition (*C*′) to the hypotheses of Theorem 18 (or Theorem 19) and the limit of orbit *O*(*T*, *z*) exists, where *z* is an element in *A* satisfying (60). Then *T* has a unique fixed point.

**Proof**

Apply the proof of Theorem 18 (or Theorem 19) and Theorem 17.

## 4. Applications

In this section, we give the several fixed point results which are obtained by our results in Section 3.

### 4.1. Fixed Point Results on an Ordinary Metric Space

Setting *α*(*x*, *y*) = *β*(*x*, *y*) = 1 for all *x*, *y* ∈ *X* in Theorem 14 (or Theorem 18), we get the following result.

**Corollary 21**

Let (*X*, *d*) be a complete metric space and *T* : *X* → *X* a continuous generalized weakly contraction mapping. Then *T* has a fixed point in *X*.

By using Remark 5, we obtain the following results.

**Corollary 22**

Let (*X*, *d*) be a complete metric space and *T* : *X* → *X* a continuous mapping and

(87) $$\begin{array}{c} d\left(Tx,Ty\right)\leq km\left(x,y\right), \end{array}$$

for all *x*, *y* ∈ *X*, where *k* ∈ [0,1) and

(88) m(x,y)=max⁡⁡{d(x,y),d(x,Tx),d(y,Ty),      12(d(x,Ty)+d(y,Tx))}.

Then *T* has a fixed point in *X*.

**Corollary 23**

Let (*X*, *d*) be a complete metric space and *T* : *X* → *X* a continuous mapping and

(89) $$\begin{array}{c} d\left(Tx,Ty\right)<m\left(x,y\right), \end{array}$$

for all *x*, *y* ∈ *X*, where

(90) m(x,y)=max⁡⁡{d(x,y),d(x,Tx),d(y,Ty),      12(d(x,Ty)+d(y,Tx))}.

Then *T* has a fixed point in *X*.

**Corollary 24**

Let (*X*, *d*) be a complete metric space and let *T* : *X* → *X* be a continuous mapping and

(91) $$\begin{array}{c} \psi \left(d\left(Tx,Ty\right)\right)\leq m\left(x,y\right)-\max\left\{d\left(x,y\right),d\left(y,Ty\right)\right\}, \end{array}$$

for all *x*, *y* ∈ *X*, where

(92) m(x,y)=max⁡⁡{d(x,y),d(x,Tx),d(y,Ty),      12(d(x,Ty)+d(y,Tx))}.

Then *T* has a fixed point in *X*.

### 4.2. Fixed Point Results on Metric Spaces Endowed with an Arbitrary Binary Relation

In this section, we give the existence of fixed point theorems on a metric space endowed with an arbitrary binary relation. Before presenting our results, we give the following notions and definitions.

**Definition 25**

Let *X* be a nonempty set and *R* a binary relation over *X*. One says that *T* : *X* → *X* is a* comparative mapping with respect to R* if

(93) $$\begin{array}{c} x,y\in X,\;xRy⟹\left(Tx\right)R\left(Ty\right). \end{array}$$

**Definition 26**

Let *X* be a nonempty set and *R* a binary relation over *X*. One says that *X* has a* transitive property with respect to R* if

(94) $$\begin{array}{c} x,y,z\in X,\;xRy,yRz⟹xRz. \end{array}$$

**Definition 27**

Let (*X*, *d*) be a metric space and *R* a binary relation over *X*. A mapping *T* : *X* → *X* is said to be a* generalized weakly contraction with respect to R* if for each *x*, *y* ∈ *X* for which *xRy* one has

(95) ψ(d(Tx,Ty))≤ψ(m(x,y)) −ϕ(max⁡⁡{d(x,y),d(y,Ty)}),

where

(96) m(x,y)=max⁡⁡{d(x,y),d(x,Tx),d(y,Ty),      12(d(x,Ty)+d(y,Tx))},

*ψ* : [0, *∞*)→[0, *∞*) is altering distance function, and *ϕ* : [0, *∞*)→[0, *∞*) is a continuous function with *ϕ*(*t*) = 0 if and only if *t* = 0.

**Theorem 28**

Let (*X*, *d*) be a metric space and *R* a binary relation over *X* and *T* : *X* → *X* a generalized weakly contraction with respect to *R*; then the following conditions hold:

(A) *T* is continuous;
(B) *X* has a transitive property with respect to *R*;
(C) *T* is comparative mapping with respect to *R*;
(D) there exists *x*_0_ ∈ *X* such that *x*_0_ *R*(*Tx*_0_).

Then *T* has a fixed point in *X*.

**Proof**

Consider two mappings *α*, *β* : *X* × *X* → [0, *∞*) defined by

(97) $$\begin{array}{c} \alpha \left(x,y\right)=\beta \left(x,y\right)=\{\begin{array}{cc} 1 & \text{if}\;xRy;\\ 0 & \text{otherwise}. \end{array} \end{array}$$

From condition (D), we get *α*(*x*_0_, *Tx*_0_) = *β*(*x*_0_, *Tx*_0_) = 1. Since *T* is comparative mapping with respect to *R*, we get *T* is *α*-admissible and *β*_0_-subadmissible. Also, *α* is forward transitive and *β* is 0-backward transitive since *X* has a transitive property with respect to *R*. Since *T* is a generalized weakly contraction with respect to *R*, we have, for all *x*, *y* ∈ *X*,

(98) ψ(d(Tx,Ty))  ≤β(x,y)ψ(m(x,y))   −α(x,y)ϕ(max⁡⁡{d(x,y),d(y,Ty)}),α(x,y)ψ(d(Tx,Ty))  ≤β(x,y)ψ(m(x,y))   −ϕ(max⁡⁡{d(x,y),d(y,Ty)}).

This implies that *T* is generalized *α*-*β*-weakly contraction mapping types *A* and *B*. Now all the hypotheses of Theorem 14 (or Theorem 18) are satisfied and thus the existence of the fixed point of *T* follows from Theorem 14 (or Theorem 18).

In order to remove the continuity of *T*, we need the following condition:

(*C*_*R*_) if {*x*_*n*_} is the sequence in *X* such that *x*_*n*_ *Rx*_*n*+1_ for all *n* ∈ *N* and it converges to the point *x* ∈ *X*, then *x*_*n*_ *Rx* for all *n* ∈ *N*.

**Theorem 29**

Let (*X*, *d*) be a metric space and *R* a binary relation over *X* and *T* : *X* → *X* a generalized weakly contraction with respect to *R*; then the following conditions hold:

(A) the condition (*C*_*R*_) holds on *X*;
(B) *X* has a transitive property with respect to *R*;
(C) *T* is comparative mapping with respect to *R*;
(D) there exists *x*_0_ ∈ *X* such that *x*_0_ *R*(*Tx*_0_).

Then *T* has a fixed point in *X*.

**Proof**

The result follows from Theorem 15 (or Theorem 19) by considering the mappings *α* and *β* given by (97) and by observing that condition (*C*_*R*_) implies property (*C*).

To assure the uniqueness of the fixed point, we will add the following condition:

(*C*_*R*_′) for all *x*, *y* ∈ *X* there exists *z* ∈ *X* such that
  (99) $$\begin{array}{c} xRz,\;\;yRz. \end{array}$$

**Theorem 30**

By adding condition (*C*_*R*_′) to the hypotheses of Theorem 28 (or Theorem 29) and the limit of orbit *O*(*T*, *z*) exists, where *z* is an element in *X* satisfying (99). Then *T* has a unique fixed point.

**Proof**

The result follows from Theorem 17 (or Theorem 20) by considering the mappings *α* and *β* given by (97) and by observing that condition (*C*_*R*_′) implies property (*C*′).

### 4.3. Fixed Point Results on Metric Spaces Endowed with Graph

Throughout this section, let (*X*, *d*) be a metric space. A set {(*x*, *x*) : *x* ∈ *X*} is called a diagonal of the Cartesian product *X* × *X* and is denoted by Δ. Consider a graph *G* such that the set *V*(*G*) of its vertices coincides with *X* and the set *E*(*G*) of its edges contains all loops; that is, Δ⊆*E*(*G*). We assume *G* has no parallel edges, so we can identify *G* with the pair (*V*(*G*), *E*(*G*)). Moreover, we may treat *G* as a weighted graph by assigning to each edge the distance between its vertices. A graph *G* is connected if there is a path between any two vertices.

In this section, we give the existence of fixed point theorems on a metric space endowed with graph. Before presenting our results, we give the following notions and definitions.

**Definition 31**

Let (*X*, *d*) be a metric space endowed with a graph *G* and *T* : *X* → *X* mapping. One says that *T preserves edges of G* if

(100) $$\begin{array}{c} x,y\in X,\;\left(x,y\right)\in E\left(G\right)⟹\left(Tx,Ty\right)\in E\left(G\right). \end{array}$$

**Definition 32**

Let (*X*, *d*) be a metric space endowed with a graph *G* and *T* : *X* → *X* mapping. One says that *X* has a* transitive property with respect to graph G* if

(101) $$\begin{array}{c} x,y,z\in X,\;\left(x,y\right)\in E\left(G\right),\\ \left(y,z\right)\in E\left(G\right)⟹\left(x,z\right)\in E\left(G\right). \end{array}$$

**Remark 33**

It is easy to see that if *G* is a connected graph, then *X* has a transitive property with respect to graph *G*.

**Definition 34**

Let (*X*, *d*) be a metric space endowed with a graph *G*. A mapping *T* : *X* → *X* is said to be a* generalized weakly contraction with respect to graph G* if for each *x*, *y* ∈ *X* for which (*x*, *y*) ∈ *E*(*G*) one has

(102) ψ(d(Tx,Ty))≤ψ(m(x,y)) −ϕ(max⁡⁡{d(x,y),d(y,Ty)}),

where

(103) m(x,y)=max⁡{d(x,y),d(x,Tx),d(y,Ty),      12(d(x,Ty)+d(y,Tx))},

*ψ* : [0, *∞*)→[0, *∞*) is altering distance function, and *ϕ* : [0, *∞*)→[0, *∞*) is a continuous function with *ϕ*(*t*) = 0 if and only if *t* = 0.

**Example 35**

Let (*X*, *d*) be a metric space, *T* : *X* → *X* a given mapping, *ψ* : [0, *∞*)→[0, *∞*) an arbitrary altering distance function, and *ϕ* : [0, *∞*)→[0, *∞*) an arbitrary continuous function with *ϕ*(*t*) = 0 if and only if *t* = 0. If *ϕ*(*d*(*x*, *Tx*)) ≤ *ψ*(*d*(*x*, *Tx*)) for all *x* ∈ *X*, then *T* is trivially generalized weakly contraction with respect to graph *G*, where *G* = (*V*(*G*), *E*(*G*)) = (*X*, Δ).

**Theorem 36**

Let (*X*, *d*) be a metric space endowed with a graph *G* and *T* : *X* → *X* a generalized weakly contraction with respect to graph *G*; then the following conditions hold:

(A) *T* is continuous;
(B) *X* has a transitive property with respect to graph *G*;
(C) *T* preserves edges of *G*;
(D) there exists *x*_0_ ∈ *X* such that (*x*_0_, *Tx*_0_) ∈ *E*(*G*).

Then *T* has a fixed point in *X*.

**Proof**

Consider two mappings *α*, *β* : *X* × *X* → [0, *∞*) defined by

(104) $$\begin{array}{c} \alpha \left(x,y\right)=\beta \left(x,y\right)=\{\begin{array}{cc} 1 & \text{if}\;\left(x,y\right)\in E\left(G\right);\\ 0 & \text{otherwise}. \end{array} \end{array}$$

From condition (D), we get *α*(*x*_0_, *Tx*_0_) = *β*(*x*_0_, *Tx*_0_) = 1. Since *T* preserves edges of *G*, we get *T* is *α*-admissible and *β*_0_-subadmissible. Also, *α* is forward transitive property and *β* is 0-backward transitive since *X* has transitive property with respect to graph *G*. Since *T* is a generalized weakly contraction with respect to graph *G*, we get

(105) ψ(d(Tx,Ty))  ≤β(x,y)ψ(m(x,y))   −α(x,y)ϕ(max⁡⁡{d(x,y),d(y,Ty)}),α(x,y)ψ(d(Tx,Ty))  ≤β(x,y)ψ(m(x,y))   −ϕ(max⁡⁡{d(x,y),d(y,Ty)}),

for all *x*, *y* ∈ *X*. This implies that *T* is generalized *α*-*β*-weakly contraction mapping types *A* and *B*. Therefore, all the hypotheses of Theorem 14 (or Theorem 18) are satisfied. Now the existence of the fixed point of *T* follows from Theorem 14 (or Theorem 18).

In order to remove the continuity of *T*, we need the following condition.

**Definition 37**

Let (*X*, *d*) be a metric space endowed with a graph *G*. One says that *X* has *G-regular property* if {*x*_*n*_} is the sequence in *X* such that (*x*_*n*_, *x*_*n*+1_) ∈ *E*(*G*) for all *n* ∈ *N* and it converges to the point *x* ∈ *X*; then (*x*_*n*_, *x*) ∈ *E*(*G*) for all *n* ∈ *N*.

**Theorem 38**

Let (*X*, *d*) be a metric space endowed with a graph *G* and *T* : *X* → *X* a generalized *α*-*β*-weakly contraction with respect to graph *G*; then the following conditions hold:

(A) *X* has *G*-regular property;
(B) *X* has a transitive property with respect to graph *G*;
(C) *T* preserves edges of *G*;
(D) there exists *x*_0_ ∈ *X* such that (*x*_0_, *Tx*_0_) ∈ *E*(*G*).

Then *T* has a fixed point in *X*.

**Proof**

The result follows from Theorem 15 (or Theorem 19) by considering the mappings *α* and *β* given by (104) and by observing that *G*-regular property implies property (*C*).

To assure the uniqueness of the fixed point, we will add the following condition:

(*C*_*G*_′) for all *x*, *y* ∈ *X* there exists *z* ∈ *X* such that
  (106) $$\begin{array}{c} \left(x,z\right)\in E\left(G\right),\;\;\left(y,z\right)\in E\left(G\right). \end{array}$$

**Theorem 39**

By adding condition (*C*_*G*_′) to the hypotheses of Theorem 36 (or Theorem 38) and the limit of orbit *O*(*T*, *z*) exists, where *z* is an element in *X* satisfying (106). Then *T* has a unique fixed point.

**Proof**

The result follows from Theorem 17 (or Theorem 20) by considering the mappings *α* and *β* given by (97) and by observing that condition (*C*_*G*_′) implies property (*C*′).

By using Remark 33, we get the following results.

**Corollary 40**

Let (*X*, *d*) be a metric space endowed with a graph *G* and *T* : *X* → *X* a generalized weakly contraction with respect to graph *G*; then the following conditions hold:

(A) *T* is continuous;
(B) *G* is connected graph;
(C) *T* preserves edges of *G*;
(D) there exists *x*_0_ ∈ *X* such that (*x*_0_, *Tx*_0_) ∈ *E*(*G*).

Then *T* has a fixed point in *X*.

**Corollary 41**

Let (*X*, *d*) be a metric space endowed with a graph *G* and *T* : *X* → *X* a generalized *α*-*β*-weakly contraction with respect to graph *G*; then the following conditions hold:

(A) *X* has *G*-regular property;
(B) *G* is connected graph;
(C) *T* preserves edges of *G*;
(D) there exists *x*_0_ ∈ *X* such that (*x*_0_, *Tx*_0_) ∈ *E*(*G*).

Then *T* has a fixed point in *X*.

**Corollary 42**

By adding condition (*C*_*G*_′) to the hypotheses of Corollary 40 (or Corollary 41), the limit of orbit *O*(*T*, *z*) exists, where *z* is an element in *X* satisfying (106). Then *T* has a unique fixed point.
